# Disability in Daily Living Activities, Family Dysfunction, and Late-Life Suicide in Rural China: A Case–Control Psychological Autopsy Study

**DOI:** 10.3389/fpsyt.2019.00827

**Published:** 2019-11-13

**Authors:** Rifang Cao, Cunxian Jia, Zhenyu Ma, Lu Niu, Liang Zhou

**Affiliations:** ^1^Department of Medical Affairs,The Seventh People’s Hospital of Hangzhou, Hangzhou, China; ^2^School of Public Health, Shandong University, Jinan, China; ^3^School of Public Health, Guangxi Medical University, Nanning, China; ^4^Department of Social Psychiatry, The Affiliated Brain Hospital of Guangzhou Medical University (Guangzhou Huiai Hospital), Guangzhou, China

**Keywords:** daily living activities, family function, suicide, older people, China

## Abstract

**Background:** Although late-life suicide in rural China is a matter of concern, research is scarce addressing the association between capability of daily living activities, family function, and late-life suicide. We conducted this psychological autopsy study to explore associations between disability in daily living activities, family dysfunction, and late-life suicide in rural China.

**Methods:** Using a 1:1 matched case–control design, we collected data from 242 elderly suicide cases and 242 living community controls in rural China using the psychological autopsy method. We used Structured Clinical Interview for the Diagnostic and Statistical Manual of Mental Disorders (SCID), Activities of Daily Living Scale (ADL), and Family Adaptive, Partnership, Growth, Affection and Resolve scale (APGAR) to assess the presence of mental disorders, capability of daily living activities, and family function, respectively.

**Results:** Compared with the living controls, suicide cases presented more severe impairment in capability of daily living activities and poorer family function. After controlling for all other factors, capability of daily living activities and family function remained in the final model. The results of the multivariate analysis also show that the presence of a mental disorder, lower levels of social support, and increased number of life events in the last year were significantly associated with elevated suicide risk.

**Conclusions:** Disability in daily living activities and family dysfunction are associated with elevated risk of late-life suicide in rural China. It is warranted to design programs, including family interventions, peer-support groups, and community programs, to help the rural elderly improve activities of daily living and obtain the social support they need to prevent suicidal behaviors.

## Introduction

Late-life suicide in rural China is a matter of concern (1). The suicide rate among people 65 years and older was six times higher than in younger age groups, and there were significantly more elderly suicides in the rural than the urban population (2). This has led the government to place greater priority on preventing late-life suicide in rural areas (3). Studies identifying the risk factors of suicide are required to design effective prevention promotion among this vulnerable population.

With aging, capability of daily living activities will decline. Studies have shown that disability in activities of daily living is related to current suicidal ideation and past suicide attempts in elderly people (4, 5). Psychosocial autopsy studies have also found that suicide decedents had more disability in activities of daily living than did living controls (6, 7). Nevertheless, evidence is scare regarding the importance of capability of daily living activities in completed suicide in elderly people in rural China.

In most cases, elderly disabled people in rural China are taken care of by a spouse or an adult child at home (8). This highlights the importance of good family function for the quality of care and quality of life of rural elderly people (9). Previous studies have linked family dysfunction with poorer health status (both physical and mental health) and worsened quality of life (10, 11), which are considered risk factors for late-life suicide (12, 13). Family dysfunction has also been found to be associated with suicidality among adolescents (14, 15). There is, however, a lack of studies focused on the relationship between family function and completed suicide in older people.

Using a 1:1 matched case–control design, this psychological autopsy study aims to investigate capability of daily living activities and family function preceding death among elderly suicide cases and to explore associations between disability in daily living activities, family dysfunction, and late-life suicide in rural China.

## Materials and Methods

### Subjects

In this study, 242 elderly suicides (aged 60 years and over) were identified from 12 counties of Shandong Province, Hunan Province, and Guangxi Province from June 2014 to September 2015. For each suicide case, one living control person with the same gender and birth year (±3 years) was randomly chosen from the same village for each suicide case. Two independent interviews with the next of kin and other individuals who were familiar with the subjects were conducted. Data from these interviews were aggregated for analysis following these principles: a) For demographic information, we basically relied on answers by the next of kin of the subjects, and b) for other risk factors, we recorded a symptom/problem as present if it was endorsed by either informant. The details of the subjects’ selection and interview procedures have been described in previous publications (16).

The institutional review boards of Shandong University, Central South University, and Guangxi Medical University approved this psychological autopsy study. Written informed consent for the publication of data and any accompanying data were obtained from the next of kin of each suicide case and the living control.

### Measures

#### Socio-Demographic Characteristics

Socio-demographic characteristics including gender, age, educational background, marital status, employment status, family annual income, living arrangement, and serious/chronic physical illness were collected. People that were married and living with the spouse or cohabiting were classified as “currently married,” while people with other marital status (including single, divorced, widowed, and married but living apart) were classified as “non-currently married.”

#### Mental Disorders

The Chinese version of the Structured Clinical Interview for the Diagnostic and Statistical Manual of Mental Disorders, Fourth Edition (DSM-IV; SCID) was used to generate current diagnoses of mental disorder (17). Diagnoses were made by psychiatrists in consensus meetings in which all information was presented, including SCID interviews with both informants and previous medical records.

#### Capability of Daily Living Activities

Capability of daily living activities was measured *via* the Activities of Daily Living (ADL) Scale (18). It consists of 14 items related to physical and instrumental activities of daily living. Item responses range from 1 (*able to perform an activity independently*) to 4 (*unable to perform an activity by oneself*). The total score ranges from 14 to 56, with higher scores indicating worse functioning of daily living activities. Elderly people were classified into three categories based on the total ADL scores: normal (≤15), mildly impaired (16–22), and severely impaired (≥22) capability of daily living activities (18).

#### Family Function

Family function was measured by the Family Adaptive, Partnership, Growth, Affection and Resolve (APGAR) scale (19). This is a validated and reliable scale that was developed to assess family function among people aged >18 years. It consists of five items rated on a three-point Likert scale ranging from 0 (*seldom*) to 2 (*often*). The total score ranges from 0 to 10, with higher scores indicating higher levels of family function. Elderly people were classified into three categories based on the total APGAR scores: low (≤3), moderate (4–6), and good (≥7) family function.

#### Social Support

Social support was measured by the 23-item Duke Social Support Index (DSSI) (20). It consists of three subscales: social interaction, subjective social support, and instrumental social support. The total score ranges from 11 to 45, with higher scores indicating higher levels of social support.

#### Life Events

Stressful life events in the last 12 months before death or investigation were measured by the Life Events Scale for the Elderly (LESE), which was developed specifically for older adults in China and consist of 46 items covering health, family, and social related life events (21).

### Data Combination

We combined the information provided by the two informants as the proxy data for each suicide case and living control. Regarding socio-demographic data, we relied on the information provided by the first informant. For psychosocial factors (i.e., capability of daily living activities, family function, social support, and life events), we chose the positive values from the two informant to integrate proxy data, taking into consideration any targeting symptoms or behaviors if observed by either informant.

### Statistical Analyses

Odds ratios (ORs) for committing suicide were calculated using conditional logistic regression. All factors were included in the multivariate analysis (enter). All analyses were performed with SPSS version 23.0 (SPSS Inc., Chicago, IL, USA). The significance levels of this study were set at 0.05.

## Results

Background characteristics are summarized in **Table 1**. Over one-third of the suicide cases and 15.3% of the living controls had severely impaired capability of daily living activities. The family function of less than one-third of the suicide cases was considered as good, while the proportion in the living controls was over 60%.

**Table 1:** Characteristics of suicide cases and living controls.

Variables	Suicide cases (*n* = 242)	Living controls (*n* = 242)	OR (95%CI)	*P*	*a*OR (95% CI)	*P*
**Gender**						
Male	135 (55.8)	135 (55.8)	1		1	
Female	107 (44.2)	107 (44.2)	1.0 (0.8–1.3)	1.000	1.1 (0.8–1.4)	0.681
**Age** [mean (SD)]		74.1 (8.2)				
60–69	73 (30.2)	70 (28.9)	1		1	
70–79	100 (41.3)	110 (45.5)	0.9 (0.7–1.2)	0.374	0.8 (0.6–1.1)	0.135
≥80	69 (28.5)	62 (25.6)	0.9 (0.6–1.3)	0.508	0.7 (0.5–1.1)	0.083
**Education**						
Below primary school	111 (45.9)	96 (39.7)	1		1	
Primary school	105 (43.4)	116 (47.9)	0.9 (0.7–1.2)	0.374	0.9 (0.7–1.3)	0.651
Above primary school	26 (10.7)	30 (12.4)	0.9 (0.6–1.3)	0.508	1.1 (0.7–1.8)	0.699
**Marital status**						
Currently married	122 (50.4)	170 (70.2)	1		1	
Non-currently married	120 (49.6)	72 (29.8)	1.5 (1.2–1.9)	0.002	1.3 (0.9–1.8)	0.191
**Employment**						
Employed	41 (16.9)	61 (25.2)	1		1	
Unemployed	194 (80.2)	167 (69.0)	1.3 (0.9–1.9)	0.091	1.1 (0.8–1.6)	0.539
Retired	7 (2.9)	14 (5.8)	0.8 (0.4–1.8)	0.647	0.9 (0.4–1.9)	0.749
**Family annual income (RMB)**						
<3600	86 (35.5)	74 (30.6)	1		1	
3,600–10,000	90 (37.2)	98 (40.5)	0.9 (0.7–1.2)	0.443	0.9 (0.7–1.4)	0.993
>10,000	66 (27.3)	70 (28.9)	0.9 (0.7–1.2)	0.532	1.0 (0.7–1.4)	0.968
**Living alone**						
Yes	64 (26.4)	35 (14.5)	1		1	
No	178 (73.6)	207 (85.5)	1.4 (1.1–1.9)	0.021	1.2 (0.8–1.7)	0.301
**Being left behind**						
Yes	41 (16.9)	25 (10.3)	1		1	
No	201 (83.1)	217 (89.7)	1.3 (0.9–1.8)	0.135	0.9 (0.6–1.2)	0.264
**Mental disorders**						
Yes	122 (50.4)	12 (5.0)	1		1	
No	120 (49.6)	230 (95.0)	2.7 (2.1–3.4)	<0.001	2.0 (1.5–2.6)	<0.001
**Physical illness**						
Yes	40 (16.5)	81 (33.5)	1		1	
No	202 (83.5)	161 (66.5)	1.7 (1.2–2.4)	0.003	1.2 (0.8–1.7)	0.338
**Daily living ability**						
Normal (≤15)	85 (35.1)	136 (56.2)	1		1	
Mildly impaired (16–21)	71 (29.3)	69 (28.5)	1.3 (0.9–1.8)	0.085	1.3 (0.9–1.8)	0.177
Severely impaired (≥22)	86 (35.5)	37 (15.3)	1.8 (1.4–2.5)	<0.001	1.4 (1.1–2.0)	0.049
**Family function**						
Good (7–10)	69 (28.5)	149 (61.6)	1		1	
Moderate (4–6)	77 (31.8)	65 (26.9)	1.7 (1.2–2.4)	< 0.001	1.2 (0.8–1.7)	0.317
Low (0–3)	96 (39.7)	28 (11.6)	2.4 (1.2–2.4)	0.001	1.5 (1.1–2.1)	0.039
**Social support**						
Highest tertile (≥28)	46 (19.0)	118 (48.8)	1		1	
Middle tertile (23–27)	89 (36.8)	76 (31.4)	1.9 (1.4–2.7)	<0.001	1.5 (1.1–2.2)	0.026
Lowest tertile (≤22)	107 (44.2)	48 (19.8)	2.5 (1.7–3.5)	0.002	1.6 (1.1–2.4)	0.015
**The occurrence of recent life events**						
No	161 (66.5)	193 (79.8)	1		1	
Yes	81 (33.5)	49 (20.2)	1.4 (1.1–1.8)	0.021	1.2 (0.9–1.6)	0.241
**Number of stressful life events in last year**						
0–1	4 (1.7)	43 (17.8)	1		1	
2–3	170 (70.2)	156 (64.5)	6.1 (2.3–16.5)	<0.001	2.9 (1.1–8.4)	0.044
≥4	68 (28.1)	43 (17.8)	7.2 (2.6–19.7)	0.002	3.7 (1.3–10.2)	0.012

Univariate analyses showed that the suicide cases were more likely to be non-currently married, live alone, and have mental disorders and physical illnesses, severely impaired capability of daily living activities, poorer family function, and lower levels of social support than the living controls. The life events with significantly increased ORs for suicide are also reported in **Table 1**.

In addition, capability of daily living activities and family function remained in the final multivariate model. The results of the multivariate analyses also show that the presence of mental disorders, lower levels of social support, and an increased number of life events in the last year were significantly associated with elevated suicide risk (**Table 1**).

## Discussion

This psychological autopsy study was conducted to shed light on the associations between capability of activities of daily living, family function, and late-life suicide in rural China. Compared with the living controls, suicide cases presented more severe impairment in capability of daily living activities, as has been reported by others (6, 7). The association between capability of daily living activities and suicide was still significant after controlling all other factors, while physical illness did not remain in the final model. This suggests that capability of activities of daily living provided a more powerful predictor of suicide than the presence of physical illness. According to the interpersonal theory of suicide (22), elderly people with greater disability in activities of daily living are more likely to perceive themselves as a burden on their families, as they are unable to bring in income and they need to be taken care by others, thereby increasing the economic burden of their family (5). This inability may cause hopelessness and consequently lead to suicide (22).

In the present study, poorer family function was significantly associated with completed suicide in the rural elderly. As discussed, elderly disabled people in rural China are usually taken care of by the spouse or an adult child at home (8). Our results showed that half of the suicide cases were single or widowed, 26.4% were living alone, and 16.9% were left behind by their adult children, which are significantly higher figures than in the living controls. It is not surprising that family dysfunction was more prevalent in the suicide cases. In addition to perceived burdensomeness, family dysfunction may raise thwarted belongingness and increase vulnerability to suicide among the disabled elderly (22). Consistent with previous findings (23), we also found that elderly people with lower levels of social support were more likely to commit suicide. Taken together, these findings underscore the need to develop community support systems for the elderly in rural China, especially to permit the disabled who lack family support to take care of themselves.

Consistent with the substantial body of published work that reports a link between mental disorder and suicidal behaviors (22, 24, 25), the presence of mental disorders was overrepresented in the suicides compared with the control group (50.4% vs. 5.0%). Additionally, presence of a mental disorder remained a factor in the final regression model. This highlights the importance of mental disorders in suicide risk and the need to improve programs to detect and treat mental disorders in rural China, where there are limited resources of mental health care.

Previous studies have also consistently shown that stressful life events commonly precede suicide (26–28). In the present study, we found that the occurrence of recent stressful life events was more common in the suicide cases than in the living controls. In addition, the number of negative life events an elderly person had experienced in the past year was a stronger predictor for suicide than the occurrence of recent events for the elderly. Further in-depth study assessing clustering of co-occurring circumstances and cumulate effects of life stressors in late-life suicide is warranted (26).

This present study has some limitations. Though psychological autopsy is a widely used method to explore the risk factors of suicide, there are methodological limitations, of which the most commonly adduced concerns the validity of data provided by proxy informants. Specifically, one set of informants was discussing an individual who had passed away 2–6 months before, while the other set of informants was discussing an individual with whom they had interacted within the past few days. Moreover, when using proxy-based information to compare suicide cases with living controls, the asymmetrical status of grief may influence the responses by the informants of difference groups. One method to reduce these differences is to use accidental death decedents or recent suicide attempters as controls (25), but this method has the potential disadvantage of obscuring the importance of risk factors that are common to completed suicide, accidental deaths, and suicidal attempts.

Despite these limitations, the data demonstrated that disability in daily living activities and family dysfunction constitute risk factors for late-life suicide in rural China. It is warranted to design programs, including family interventions, peer-support groups, and community programs, to help the rural elderly (in particular those non-currently married, living alone, and left behind) improve their activities of daily living and obtain the social support they need to prevent suicidal behaviors.

## Data Availability Statement

The raw data supporting the conclusions of this manuscript will be made available by the authors, without undue reservation, to any qualified researcher.

## Ethics Statement

The studies involving human participants were reviewed and approved by Central South University. The patients/participants provided their written informed consent to participate in this study.

## Author Contributions

RC conducted the data analysis and drafted the manuscript. CJ, ZM, LN, and LZ contributed to the study design and provided substantial editorial input in the drafting of the manuscript. All authors read and approved the final manuscript.

## Funding

This work was supported by the American Foundation of Suicide Prevention to LZ (grant no. SRG-0-169-12).

## Conflict of Interest

The authors declare that the research was conducted in the absence of any commercial or financial relationships that could be construed as a potential conflict of interest.
